# Galvanism

**Published:** 1841-12

**Authors:** George G. Brewster

**Affiliations:** Portsmouth, N. H.


### ARTICLE V.

*Galvanism.* By GEORGE G. BREWSTER, ESQ. Portsmouth, N. H.

MESSRS. EDITORS:—I have carefully examined the contents of Mr. Brockway's paper, published in your valuable periodical (lib. i, fol. 127) relative to the discovery he presumes himself to have made, and which inclines him to aver, "that the present method of attaching teeth to gold plate ought not to be continued; the different metals combined converts the mouth into a galvanic battery."

To enter into the *multimodus operandi* of galvanic action, for the purpose of disproving the views expressed by that gentleman, would require much time in the preparation, and occupy a greater number of pages in the Journal than could be properly afforded, or the merits of the theme could justly demand.

A philosophical discussion could go no farther than to prove that the nature of the subject of galvanism is of no greater imwith ossific matter is denied by some; but the writer of this has met with several instances, as was clearly ascertained after the extraction of the teeth, where depositions of bone had actually taken place, and in five or six cases, in considerable quantities. He has also conversed with several of his professional brethren, who say they have observed the same thing, and among the number, Dr. H. H. Hayden, of this city, a gentleman whose scientific attainments and professional acumen would render any deception in the matter altogether improbable.

The establishing of this fact, though somewhat irrelevant to the subject under discussion, was deemed necessary, in order to show the morbid effects that are frequently induced in the gum and socket of a superior molaris, by the loss of its antagonist.

portance to the science of dental surgery, than magnetism, electricity, or even astronomy. It may be considered but in common with the laws of nature, and as the mere result of a chemical action.

I shall be laconic in this treatise, presuming every intelligent practitioner of dentistry will be satisfied with being reminded of the principles of galvanism, so far only as have the nearest relation to the views presented in Mr. Brockway's vague theory, and presenting for the consideration of those who give credence to that gentleman's doctrine, a few of the many cases which have come under observation since he introduced his novel ideas to your readers.

It must be self-evident to any person having a tolerable acquaintance with the nature of galvanism, that according to Mr. Brockway's description of that which he conceives to be galvanic action, there could not have been a perceptible effect of voltaic character, beyond that which he felt in the region of his philosophizing imagination. Ascribing the "several successive shocks," felt by his patients to galvanic action, could, with as much propriety have been attributed to any other cause, and certainly to that of a mechanical, with much more rationality.

The subject does not call for discussion, until attempts are made to establish the novel ideas advanced in Mr. Brockway's hypothesis ; and until then it may not be unsafe to hold as an established principle in the laws of nature, "that galvanism has no farther action than there is a chemical decomposition of its most effective elements." And as an inference and natural consequence, that a long series of galvanic action must so result in the change and waste of particles of its elements as to give occular proof of the degree of corrosion which has been produced in the operation, and in that degree which will show plainly the reduction of size of the most corrosible parts acted upon. If there has not been a chemical change in the elements of galvanism, there is no proof of the production of galvanic action, even in a development of most subtile character.

Since Mr. Brockway published his hypothesis on "*Galvanic action of metal in the mouth,*" I have had several opportunities for examining soldered gold plates which had been in use during a long time, and were brought to my rooms for alterations.

Among others there was one case of a plate which I made for the lady of the late Judge L****e, in 1829. The teeth were mineral and confined to the plate with soldered gold pivots; the springs embracing the two first superior molares were likewise confined to the plate with gold solder. Before polishing the plate, I reduced the projecting parts of the solder to a surface even with the plate, and when the whole was finished, it presented an appearance as if there had not been a perforation through the plate, or that solder had been applied. Twelve years constant use of this plate and its solder had elapsed when the lady returned to my room (being the third time after the teeth were inserted) I entered into a careful examination of the condition of the metals, and found that during that time there had not been so much corrosion of the solder as to have occasioned the loss of the least discoverable particle of its substance exceeding that from the surface of the main body of the gold. It had, however, become more dim than the plate; but when I had brushed the plate and solder on all their parts to an equal brightness, I could not discover that the solder had suffered in the least degree of chemical action beyond that which the plate had undergone.

Within a few weeks, a gentleman called upon me to set a mineral tooth, and make several corrections to a full set of teeth made in Doctor Flagg's room, in Boston. They are of fine workmanship, and had been in operation about two years. I paid particular attention to the state of the solder, and although I think it was not of as many carats fine as that mentioned in the first case, yet I could detect no farther chemical change than simply that the form of the solder showed itself by a slight tarnish, rather darker than the plates and springs. By the mere application of the brush and the common process for cleaning the plates, the whole oxidation was removed. I could not discover any greater decomposition or waste of particles of the solder than of the plates of gold.

Mrs. G. of Exeter, N. H. three years since employed an itinerant, who frequently stops in the vicinity of her residence, for the purpose of "*mending teeth and fixing in new ones.*" She applied to me lately and requested there might be an adjustment of the wires and plate of teeth which he set for her. On examination I found the plate and teeth pendent from the superior *bicuspides* with springs surrounding. The various metallic parts were held together by *silver solder.* The chemical result was that it had been changed to a dark hue and an evident decomposition had taken place on the surfaces of the various parts of the solder, which presented an appearance similar to silver which had been oxidized by being heated in contact with sulphur. When scraped with a steel instrument the particles thrown off appeared like plumbago.

I have here placed for consideration a few cases, of the many similar, which have come under observation in the course of my late practice. How, agreeably to the known principles of galvanism, Mr. Brockway, Dr. Mackall, and other intelligent practitioners in the profession, whose scientific acquirements and philosophical mode of reasoning would seem sufficient to enlighten the community of dental surgeons, on a subject which thus far has been treated in our profession, in a mere vague and opiniative manner, can suffer so important a branch of science to the cause of dentistry, as they deem galvanism to be, to pass without an explanation of its principles, and hold forth ideas without argument and inconsistent with the laws of nature; will talk of galvanic action producing *shocks, pains, decomposition of the teeth, absorption of the alveolar process,* and a host of miseries to be increased only proportionate with a galvanic excitement of the practitioner's notions; is what I understand no farther of, than that they ascribe to galvanism a power, which, with such elements as they mention, is increased more than six thousand times, in their descriptions of effects, beyond their capacities to prove by any philosophical experiment, or proved theory.*

* That a galvanic action is produced by placing two or more different kinds of metal in the mouth, is to us, not only in strict accordance with the laws of galvanism, but is also supported by facts of almost daily occurrence. All, however, are not equally susceptible to, or conscious of its action, nor are results as pernicious as those represented by Mr. Brockway, often produced by it; and until some better method than that which he proposes be invented, we shall continue the present mode of attaching teeth to the plate. If the gold and the solder be sufficiently pure, and the necessary attention to the cleanliness of the mouth be observed, artificial teeth set upon plate, may be worn with impunity. Mr. Brockway's plan does not obviate the objection urged by him, for in attaching the clasp to the plate, solder must be employed, and furthermore, his method of securing the teeth to the plate is such as to afford no guarantee that they will remain for any considerable length of time.

